# Glutamatergic Dysfunction and Glutamatergic Compounds for Major Psychiatric Disorders: Evidence From Clinical Neuroimaging Studies

**DOI:** 10.3389/fpsyt.2018.00767

**Published:** 2019-01-24

**Authors:** Cheng-Ta Li, Kai-Chun Yang, Wei-Chen Lin

**Affiliations:** ^1^Department of Psychiatry, Taipei Veterans General Hospital, Taipei, Taiwan; ^2^Institute of Brain Science and Brain Research Center, National Yang-Ming University, Taipei, Taiwan; ^3^Division of Psychiatry, Faculty of Medicine, National Yang-Ming University, Taipei, Taiwan; ^4^Institute of Cognitive Neuroscience, National Central University, Jhongli, Taiwan

**Keywords:** glutamate, major psychiatric disorders, NMDA antagonist, antidepressant, neuroimaging

## Abstract

Excessive glutamate release has been linked to stress and many neurodegenerative diseases. Evidence indicates abnormalities of glutamatergic neurotransmission or glutamatergic dysfunction as playing an important role in the development of many major psychiatric disorders (e.g., schizophrenia, bipolar disorder, and major depressive disorder). Recently, ketamine, an *N*-methyl-d-aspartate antagonist, has been demonstrated to have promisingly rapid antidepressant efficacy for treatment-resistant depression. Many compounds that target the glutamate system have also become available that possess potential in the treatment of major psychiatric disorders. In this review, we update evidence from recent human studies that directly or indirectly measured glutamatergic neurotransmission and function in major psychiatric disorders using modalities such as magnetic resonance spectroscopy, positron emission tomography/single-photon emission computed tomography, and paired-pulse transcranial magnetic stimulation. The newer generation of antidepressants that target the glutamatergic system developed in human clinical studies is also reviewed.

## Introduction

### Overview of Glutamate and Risks of Neuropsychiatric Disorders

Glutamate is the most abundant excitatory neurotransmitter in the human brain and has critical roles in multiple brain functions and synaptic plasticity, such as long-term potentiation. However, excessive glutamate release can be toxic to the brain and has been linked to many neurodegenerative diseases, such as Alzheimer's disease, amyotrophic lateral sclerosis, and Huntington's disease (1). Glutamate excitotoxicity has been associated with exposure to severe stress, and excessive glutamate release and uptake have been identified in brain regions such as the frontal cortex and hippocampus of rats exposed to various forms of stress (2, 3). A growing body of evidence also indicates that abnormalities of glutamatergic neurotransmission play an important role in the development of many major psychiatric disorders (e.g., schizophrenia, bipolar disorder [BD], and major depressive disorder [MDD], including treatment-resistant depression [TRD]) (4, 5). In this review, we first update evidence from recent neuroimaging human studies that pinpoints glutamatergic dysfunction in the pathophysiology of major psychiatric disorders.

### Glutamate Receptors and Subunits

Glutamate receptors can be divided into two categories: ionotropic glutamate receptors (iGluRs) and metabotropic glutamate receptors (mGluRs) (6). iGluRs with an ion channel pore activate when glutamate binds to receptors, whereas mGluRs activate ion channels on the plasma membrane indirectly through a signaling cascade. iGluRs have three subtypes of receptors—*N*-methyl-d-aspartate (NMDA) receptors, α-amino-3-hydroxy-5-methyl-4-isoxazolepropionic acid (AMPA) receptors, and kainate receptors—based on the chemical that binds to them more selectively than glutamate. Mammalian mGluRs are categorized into three groups: group 1 (mGluR1 and mGluR5), group 2 (mGluR2 and mGluR3), and group 3 (mGluR4, mGluR6, mGluR7, and mGluR8). Activation of the NMDA receptor (NMDAR) requires a glutamate binding to its NR2 subunits and a glycine binding to its NR1 subunits. Then, a nonspecific cation channel is opened, enabling Ca^2+^ and Na^+^ to enter and K^+^ to exit the cell (7).

### Neuroimaging Techniques in Assessing Glutamate-Related Function in Human Brains

To date, it remains difficult to measure glutamatergic neurotransmission in human brains. However, some techniques have been developed to explore glutamatergic neurotransmission by calculating glutamate levels, such as magnetic resonance spectroscopy (MRS), or by measuring glutamate-related function, including positron emission tomography (PET)/single-photon emission computed tomography (SPECT) and paired-pulse transcranial magnetic stimulation (ppTMS).

MRS is a specialized technique that directly quantifies brain molecules, including glutamate. This noninvasive and ionizing radiation-free neuroimaging technique is associated with magnetic resonance imaging (MRI), and both are used to acquire signal from hydrogen protons (or other nuclei, including carbon and nitrogen).

Typically, MRS is used to measure signals within a predefined region of interest (i.e., a voxel), although the technique is still being updated to acquire biochemical signals from the whole brain (8). Using ^1^H-MRS, and depending on the chemical environment, each proton may be visualized at a specific chemical shift (peak position along the chemical shift axis). Glutamate (Glu) and glutamine can thus be marked by a series of resonance peaks between 2.2 and 2.4 ppm, which means that these metabolites overlap and are often referred to in combination as Glx. Some editing techniques (e.g., echo time averaging) enable differentiation of glutamate from glutamine (9). It has been reported that field strengths of 4 T or more are better for the separation of glutamate from glutamine (10). However, the information of glutamate derived by MRS does not exactly reflect glutamatergic neurotransmission of the neurons but rather the total level/concentration in the given voxel.

PET enables measurement of brain molecules by detecting gamma rays originating from the annihilation between electrons and positrons emitting from radionuclides. iGluRs are ligand-gated ion channels that mediate excitatory neurotransmission in human brains and can be divided into at least three groups: NMDA, AMPA, and kainate receptors (11). Several radioligands have been developed to successfully image different subtypes of NMDARs *in vivo* (11). Moreover, human studies have been conducted using PET imaging with ^11^C-ABP688, a radioligand for mGluR5, to evaluate ketamine-induced glutamate release both in healthy subjects (12) and in patients with MDD (13). However, the application of this PET imaging paradigm to measure glutamate receptors in clinical settings might be limited by the availability of the radioligand and the need for arterial input function for quantification. In addition, because PET/SPECT measures specific molecules, the findings do not represent exact glutamatergic neurotransmission. Alternatively, ^18^F-fluorodeoxyglucose (^18^F-FDG)-PET, a clinical imaging tool widely used to measure brain glucose uptake with favorable signal-to-noise ratio in most brain regions, has been proposed to be a proxy measure of glutamatergic neurotransmission (14, 15). The rationale is that glutamate is produced in neurons from glucose-derived tricarboxylic acid cycle intermediates and branched-chain amino acids. The reuptake of glutamate from the synaptic cleft is coupled with Na^+^/K^+^-ATPase activation and glucose use (16). With neuronal depolarization and glutamate being released into the synaptic cleft from presynaptic vesicles, the process requires energy and is dependent on the use of glucose. Interestingly, ketamine-induced increased glucose uptake in patients with MDD (14) is in line with decreased ^11^C-ABP688 binding in similar brain regions (13). Thus, ^18^F-FDG-PET might serve as a promising tool to evaluate glutamatergic neurotransmission.

ppTMS is a noninvasive technique that manipulates the strength and stimulus intervals between two pulses to measure cortical inhibition and excitation in humans (17, 18). It can be used to examine at least two different corticocortical inhibitory processes in the human motor cortex that are mediated by different subtypes of GABAergic receptors: short-interval cortical inhibition and long-interval cortical inhibition (19). Moreover, ppTMS can also be used to examine a corticocortical excitatory process, intracortical facilitation (ICF), when a subthreshold pulse precedes a test pulse by 8–30 ms (17, 20). The resulting facilitation of the motor-evoked potential response has been found to be mediated mainly by glutamatergic neurotransmission. When a glutamate antagonist, riluzole, is used, ICF can be suppressed without influencing cortical inhibition (21). Such findings indicate that the neurotransmitter glutamate is involved in facilitatory mechanisms of the motor cortex. Compared with the aforementioned techniques, ppTMS measurement such as ICF is more likely to reflect functional glutamatergic neurotransmission in the testing cortical region but not levels of the subtypes of glutamate receptors. In addition, I-wave facilitation is another ppTMS measurement that reflects glutamatergic activity of a different neuron population to ICF and could be mediated by non-NMDA receptors (22).

### Schizophrenia and Glutamatergic Dysfunction

Schizophrenia is a major psychiatric disorder characterized by prominent psychotic symptoms and abnormal social behaviors. Despite most current antipsychotics being dopamine antagonists or acting on dopamine receptors, alterations in glutamatergic neurotransmission could be critical to the pathophysiology of schizophrenia. For example, administration of the NMDAR antagonist phencyclidine or ketamine could induce a schizophrenia-like state in human subjects (23, 24), supporting the hypothesis that glutamatergic dysfunction plays a crucial role in the pathophysiology of schizophrenia. Furthermore, group I mGluRs are heavily expressed in basal ganglia that contain high densities of dopamine receptors (25), and at least two independent studies have identified several deleterious single-nucleotide polymorphisms (SNPs) in the human gene encoding mGluR subtype I in patients with schizophrenia (26). Despite inconsistency, some postmortem studies have also revealed that iGluRs and mGluRs are abnormally expressed in human subjects with schizophrenia. For example, iGluR-AMPA receptors and kainate receptors were decreased in expression in the schizophrenic hippocampus, and the iGluR-NMDAR subunit NR1 might be abnormally expressed in some cortical regions in schizophrenia (27), whereas higher mRNA levels for group I mGluRs were found in the prefrontal cortex (Brodmann area 9) in patients with schizophrenia (28).

A large meta-analysis of ^1^H-MRS studies identified 59 studies that included 1,686 patients and 1,451 healthy control subjects (Table 1) (29). By adopting a random-effects, inverse-weighted variance model to calculate the pooled effect size, the investigators found that, in schizophrenia, there were significant elevations in glutamate in the basal ganglia (Hedges' *g* = 0.63; 95% confidence interval [CI], 0.15–1.11), glutamine in the thalamus (Hedges' *g* = 0.56; 95% CI, 0.02–1.09), and Glx in the basal ganglia (Hedges' *g* = 0.39; 95% CI, 0.09–0.70) and the medial temporal lobe (Hedges' *g* = 0.32; 95% CI, 0.12–0.52). No regions exhibited a reduction in glutamate metabolites in schizophrenia. A systemic review pinpoints that increased Glx in many cortical regions, including prefrontal cortex, temporal cortex, parietal cortex, and occipital cortex, as well as increased glutamine in thalamus, prefrontal cortex, and anterior cingulate cortex (30). By contrast, a recent systemic review and meta-analysis of ^1^H-MRS studies on antipsychotic-naïve/free patients with schizophrenia included 21 studies and noted no changes in glutamate-related metabolites (31). Because brain glutamate levels may be confounded by drug use and related to response to antipsychotics, Egerton et al. investigated glutamate levels (Glu/Cr) in the anterior cingulate cortex and thalamus in antipsychotic-naïve or minimally medicated patients with first-episode psychosis, and they found that higher levels of glutamate in the anterior cingulate cortex were associated with more severe psychotic symptoms at presentation and a lower likelihood of being in remission 4 weeks after amisulpride treatment (32).

**Table 1:** Summary of major neuroimaging findings to support glutamatergic dysfunction in major psychiatric disorders.

	**Schizophrenia**	**Bipolar disorder**	**MDD**
MRS	↑Glutamate (BG^*^, ACC, THA)	↑Glx (PFC^*^)	↓Glx (PFC^*^, ACC^*^)
	↑Glutamine (THA^*^, PFC, ACC)	Glutamate: no change^*^	↓Glutamate (ACC^*^)
	↑Glx (BG^*^, MTL^*^, PFC, OC, PC)		
PET	↓NMDA receptor binding (HIPPO)	Lack of direct evidence	↓mGluR5 (ACC, OFC, BG, AMG, HIPPO)
	↑Dopamine uptakes (BG)	↑Glucose uptakes (BG) after ketamine	↑Glucose uptakes (PFC) after ketamine
	↑Glucose uptakes and blood flow (ACC) after ketamine		
	↑Glucose uptakes (PFC) after ketamine		
pp-TMS	↑ICF (first-episode patients)	No reports on ICF and I-wave facilitation	ICF: no difference; but
	↑I-wave facilitation		↑ICF in young MDD

*BG, Basal ganglia;ACC, anterior cingulate cortex; MTL, medial temporal lobe; THA, thalamus; PFC, prefrontal cortex; OFC, orbital frontal cortex; HIPPO, hippocampus; AMG, amygdala; PC, parietal cortex;OC, occipital cortex*.

* *Supported by at least one meta-analysis*.

Development of the glutamate hypothesis of schizophrenia was initially based on the effects of phencyclidine, which acts primarily as an NMDAR antagonist. Because presynaptic dopamine release is under the control of inhibitory GABAergic neurons that are activated by NMDARs, previous PET/SPECT studies that used D2/D3 receptor ligands (e.g., ^11^C-raclopride and ^123^I-iodobenzamide) in schizophrenia provided evidence for glutamate dysfunction in schizophrenia. A review study including D2/D3 receptor PET/SPECT studies revealed that increased dopamine uptake in the striatum and putamen was observed in schizophrenia, indicating an effect of NMDA blockade on striatal dopamine release (33). Not only findings in the basal ganglia, early studies measured cerebral blood flow following ketamine infusion in patients with schizophrenia by 15O-H2O PET and found increased blood flow in anterior cingulate cortex (34), which was found to correlate the ketamine-induced psychosis-effects in healthy control subjects (35). A study using 18F-FDG PET to study glucose metabolism after ketamine also revealed increased metabolism in frontal cortex and anterior cingulate cortex (36). In addition, repeated ketamine administration had been found to have increased dopamine D1 receptor binding in the dorsolateral prefrontal cortex by using 11C-NNC112 (37). As for studies using radioligands to image glutamatergic receptors directly, 123I-CNS-1261 as a SPECT ligand acts on NMDA receptors had been used in healthy subjects to study the binding of ketamine and the results found ketamine led to a global reduction in the binding signals (38). One study found the reduction of 123I-CNS-1261 binding after ketamine was greatest in thalamus, basal ganglia, and frontal cortex, which mainly correlated with negative symptoms (39). While marked reduction of NMDA receptor bindings had been reported in patients treated with schizophrenia, a study directly applying this compound in medication-free patients with schizophrenia found significant reductions of NMDA receptor binding in left hippocampus (40).

ICF of ppTMS reflects glutamatergic neurotransmission in the motor cortex. A meta-analysis including ppTMS studies from 1990 to 2012 found no changes of ICF, but decreased short-interval intracortical inhibition, in schizophrenia (41). By contrast, later research with small sample sizes revealed significant increases of ICF in first-episode schizophrenia compared with healthy control subjects (42). In addition, I-wave facilitation was found to be increased in patients with schizophrenia (43). Future studies with larger sample sizes controlling chronicity of illness course and the use of medications are still needed.

### Bipolar Disorder and Glutamatergic Dysfunction

BD is a major psychiatric disorder characterized by prominent mood fluctuation, including episodes of depression and periods of abnormally elevated mood. Postmortem studies of patients with BD revealed reduced expression of NMDAR subunit NR1 in the prefrontal cortex (44) and reduced expression of several NMDA, AMPA, and kainite receptor subunits in the medial temporal cortex (45), although other postmortem research found that mGluRs seem to be less involved in the anterior cingulum of patients with BD (46). However, genome-wide association studies (GWAS) and SNP results have also provided genetic evidence that glutamate signaling is implicated in the pathophysiology of BD (47, 48).

Previous neuroimaging studies in patients with BD (Table 1) have provided evidence that glutamate dysfunction plays a crucial role in the pathophysiology of BD. A meta-analysis of ^1^H-MRS studies from 1980 to 2010 on brain glutamate and glutamine in BD showed that patients with BD had widespread increased Glx, including in the prefrontal cortex, compared with healthy control subjects, although no significant difference in Glu/Cr was noted (49). The finding of increased Glx in the frontal cortex was replicated in a subsequent meta-analysis (50). However, the results to date have been inconsistent, and many factors, including mood status and medication, might affect glutamate levels. For example, a recent systemic review examined the effects of lithium, a drug commonly used for mood control, by summarizing results of 26 ^1^H-MRS studies (51). The investigators found inconclusive results regarding glutamate levels and the influence of lithium treatment.

Few PET/SPECT studies have used radioligands specifically to target glutamate receptors in BD. However, ketamine, an NMDA antagonist, has been found to have rapid antidepressant effects in depressed patients with BD and has been studied in combination with ^18^F-FDG-PET before and after ketamine infusion. That study found that brain glucose metabolism changes in the right ventral striatum of basal ganglia were significantly correlated with depression improvement (52). Because ^18^F-FDG PET in combination with glutamatergic agents could serve as a proxy for glutamate neurotransmission, the findings suggest that ketamine improved BD depression by promoting glutamatergic neurotransmission in brain regions involved in mood control. However, questions remain. For example, whether glutamate dysregulation is the fundamental cause of the pathophysiology of BD and whether glutamatergic agents can reverse the brain abnormalities of BD. We compared brain glucose metabolism in subtypes of BD by using ^18^F-FDG-PET and found that patients with BD type I (compared with patients with BD type II) had significantly lower glucose metabolism in the bilateral anterior cingulum, insula, striatum, and part of the prefrontal cortex and higher glucose metabolism in some limbic structures (53). Frontolimbic dysregulation seems to play a critical role in the pathophysiology of BD, because the unaffected siblings of patients with BD have such abnormalities in a minor form that can be detected by resting-state functional MRI but not by ^18^F-FDG-PET (54). Whether these abnormal findings are a result of glutamate dysregulation warrants further investigation. Furthermore, ppTMS research that specifically examines ICF is still lacking, despite such research demonstrating cortical inhibitory deficits in patients with BD (55).

### Major Depressive Disorder/Treatment-Resistant Depression and Glutamatergic Dysfunction

MDD is a severe psychiatric disorder characterized by episodes of depression and anhedonia. MDD is considered a severe illness because of a tendency for the illness to become chronic and a high prevalence of TRD. Evidence has revealed a pivotal role of glutamatergic neurotransmission in the pathophysiology of MDD.

Postmortem studies have revealed that expression of mGluR2/3 receptors in the anterior cingulate cortex was significantly reduced in patients with MDD (56), whereas another study found no significant difference in the anterior cingulate cortex between patients with MDD and healthy control subjects in expression of mGluR2/3 or mGluR5 (46). Furthermore, iGluRs are abnormally expressed in human subjects with MDD. For example, the expression of NMDAR subunit NR2 in the prefrontal cortex was reduced in patients with MDD (57). A study using postmortem brains of patients with MDD further demonstrated that elevated expression levels of the majority of mGluR and iGluR genes were found in the dorsolateral prefrontal cortex, and the genetic expression differences occurred mostly in female subjects (58). A meta-analysis of three large MDD GWASs (4,346 subjects with MDD vs. 4,430 control subjects) found that genes involved in glutamatergic synaptic neurotransmission were significantly associated with MDD (59).

Regarding neuroimaging findings in clinical patients (Table 1), ^1^H-MRS studies in patients with MDD have revealed decreased glutamate and glutamine levels in the dorsolateral and other parts of the prefrontal cortex and increased glutamate levels in the occipital cortex (60). A meta-analysis of ^1^H-MRS studies demonstrated that decreased Glx levels with absolute values in the prefrontal cortex were correlated with treatment severity (i.e., number of failed antidepressant treatments), indicating that the severity of glutamatergic dysregulation could be related to the severity of illness (61). Another meta-analysis noted that glutamate and Glx concentrations were lower in the anterior cingulate cortex in patients with MDD than in control subjects (62).

**Figure 1:**
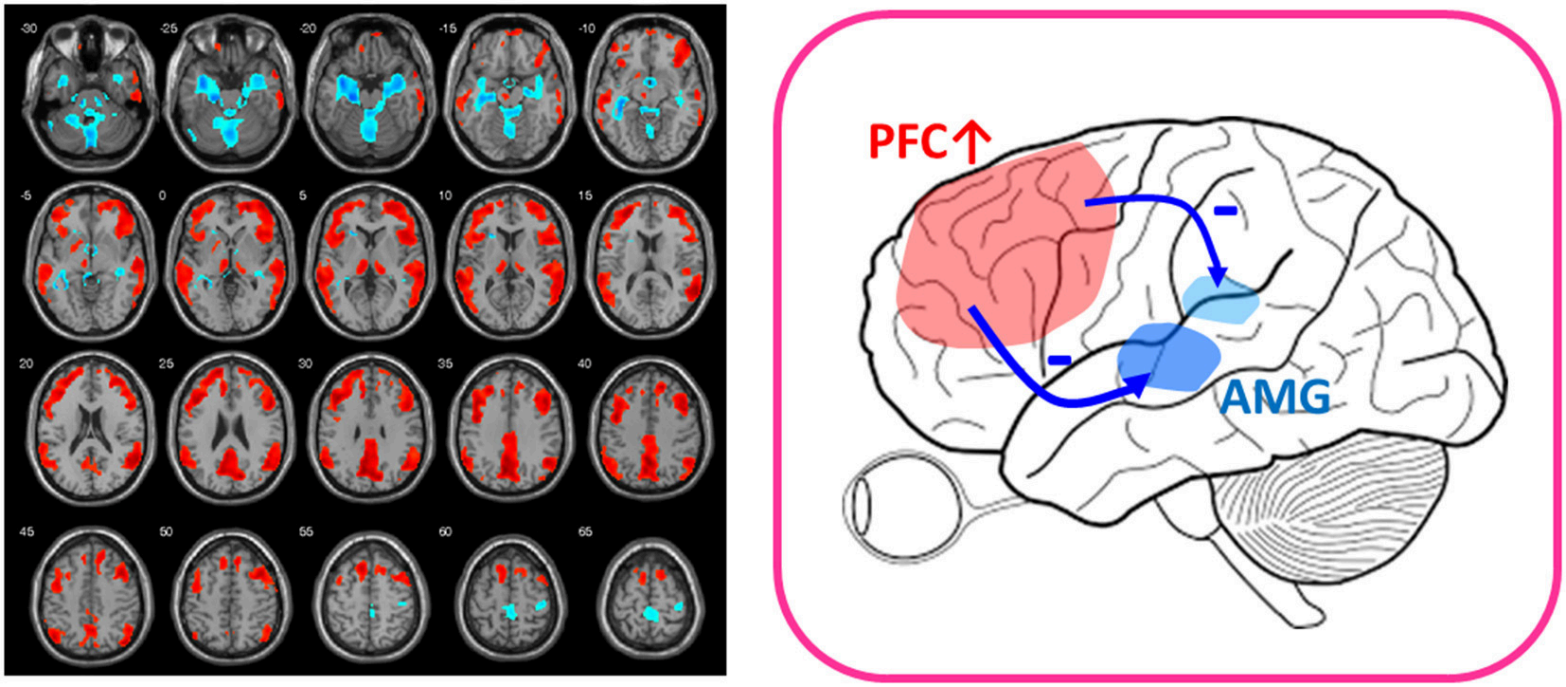
After-vs.-before changes of glucose metabolism in response to low-dose ketamine in treatment-resistant depressives (FWE-corrected, *p* < 0.001). Patients showed decreased glucose metabolism in limbic structures, such as amygdala (AMG) and hippocampus (shown in blue color) and increased function in prefrontal cortex (PFC) (shown in red color) after ketamine treatment. The placebo group lacked the PFC activation (14). The findings of prefronto-amygdala changes in response to ketamine provided supports that low-dose ketamine could reverse glutamatergic dysfunction of the mood circuit.

A further piece of evidence comes from the surprisingly rapid antidepressant response to low-dose ketamine in the treatment of TRD. Structural and functional abnormalities in the prefrontal cortex have been found to be prominent in patients with TRD (63, 64), and intravenous low-dose ketamine (0.2–0.5 mg/kg) was revealed to reverse the prefrontal abnormalities and frontolimbic dysregulation of the human brain in 1 h (14). We applied ^18^F-FDG-PET before and after intravenous injection of 0.5 mg/kg ketamine, 0.2 mg/kg ketamine, and placebo and found that prefrontal cortical function increased only in the low-dose ketamine groups, whereas the activation of prefrontal function correlated well with the deactivation of limbic function in the amygdala and hippocampus (Figure 1) (14). Moreover, recent PET research using ^11^C-ABP688, a radioligand for mGluR5, revealed a significant ketamine-induced reduction in mGluR5 availability as reflected by decreased ^11^C-ABP688 binding in all subjects, which persisted for more than 24 h (13). In addition, the changes of ^11^C-ABP688 binding were correlated with the rapid antidepressant effect of ketamine. However, because ketamine is an NMDA antagonist (one type of iGluR), future studies directly investigating iGluRs in response to low-dose ketamine are warranted.

Although some ppTMS studies have found no significant differences in ICF between patients with MDD and healthy subjects (41, 65), few studies have differentiated MDD from TRD. In addition, medication could obviously confound the ppTMS findings, given that medicated euthymic patients with MDD exhibited much increased ICF compared with healthy control subjects and unmedicated patients with MDD (65). Furthermore, patients with MDD with a comorbidity of generalized anxiety disorder are more likely to have poorer responses to antidepressants than those without (66). Our team demonstrated that patients with generalized anxiety disorder in unmedicated status had decreased ICF in bilateral motor cortices, suggesting that patients with more impaired glutamatergic neurotransmission in cortical regions may have worse treatment outcomes (5). A study targeting at child and adolescence depressives (9–17 years old) and found depressed patients had significantly increased ICF (67). Future studies that control medications and more specifically target TRD over MDD without a history of antidepressant resistance are necessary.

### Glutamate Compounds That Target Glutamatergic Dysfunction

As aforementioned, glutamate receptors can be divided into iGluRs and mGluRs (6). iGluRs include NMDARs, AMPA receptors, and kainate receptors, whereas mGluRs can be categorized into group 1, group 2 (mGluR2 and mGluR3), and group 3. More recently, selective glutamate positive allosteric modulators (PAMs) have been developed that enhance glutamate receptor function in the presence of endogenous agonists without having adverse effects resulting from intrinsic activity (e.g., NMDAR PAMs) (68). By contrast, glutamate mGluR2 or mGluR5 (basimglurant) negative allosteric modulators (NAMs) may inhibit glutamate receptor function and have been tried in MDD studies, although the results are mixed (69). Therefore, given that glutamatergic dysfunction plays a crucial role in the major psychiatric disorders discussed herein, compounds aimed at activating or inhibiting the aforementioned receptors or indirectly modulating functions of receptors are of great research interest. Table 2 lists glutamatergic compounds that have been used to treat major psychiatric disorders in human clinical trials (searched in the ClinicalTrials.gov database, accessed on September 20, 2018).

**Table 2:** Glutamatergic compounds in the treatment of major psychiatric disorders.

**Glutamatereceptors**	**Compounds**	**Mechanisms**	**Target diseases**	**Examples of ClinicalTrials.gov Identifier(C: completed; T: terminated)**
iGluR-NMDA	Ketamine	An NMDA antagonist	MDD in alcoholism	NCT01551329 (phase 1) (C)
			Bipolar depression	NCT01833897 (phase 4) (C)
	Esketamine	An NMDA antagonist	MDD (TRD)	NCT02782104 (phase 3)
			Imminent suicide risks	NCT02133001 (phase 2) (C)
	D-cycloserine	Mixed agonist/antagonist at NMDA receptor/glycine binding site	MDD (TRD)	NCT00408031 (phase 2) (C)
			Bipolar depression	NCT01833897 (phase 4) (C)
			Schizophrenia	NCT02769936 (phase 1) (C)
	D-serine	An NMDA-glycine site agonist	Schizophrenia	NCT00322023 (phase 2) (C)
	RO4917838 (Bitopertin)	A glycine reuptake inhibitor	Schizophrenia	NCT01235585 (phase 3) (C)
	NRX-101	D-cycloserine + lurasidone	Bipolar depression	NCT03395392 (phase 2)
	Riluzole	A glutamate release inhibitor	MDD (TRD)	NCT00088699 (phase 2) (C)
			Bipolar depression	NCT00054704 (phase 2) (T)
	Nitrous oxide	An NMDA antagonist	MDD (TRD)	NCT02994433 (phase 1)
	NMDAE	An NMDA enhancer	MDD	NCT03414931 (phase 2) (C)
	Nuedexta	Dextromethorphan+quinidine	MDD (TRD)	NCT01882829 (phase 2) (C)
		Dextromethorphan as an NMDA antagonist		
	AXS-05	Dextromethorphan+bupropion	MDD (TRD)	NCT02741791 (phase 3)
		Dextromethorphan as an NMDA antagonist		
	CP-101,606 (traxoprodil)	An NMDA receptor subunit GluN2B Antagonist	MDD (TRD)	NCT00163059 (phase 2) (C)
	Memantine	An NMDA antagonist	MDD	NCT00040261 (phase 3) (C)
	Sarcosine	An NMDA enhancing agent (a glycine transporter-I inhibitor)	MDD	NCT00977353 (phase 2) (C)
			Schizophrenia	NCT01503359 (phase 2) (C)
	AZD6765	An NMDA channel blocker	MDD (TRD)	NCT00986479 (phase 2) (C)
	CERC-301	An NMDA GluN2B antagonist	MDD	NCT02459236 (phase 2) (C)
	MK-0657	A selective NMDA GluN2B antagonist	MDD (TRD)	NCT00472576 (phase 2) (C)
	NRX-1074	An NMDA partial agonist	MDD	NCT02067793 (phase 2) (C)
	GLYX-13 (Rapastinel)	An NMDA receptor enhancer	MDD (TRD)	NCT01684163 (phase 2) (C)
	REL-1017 (d-Methadone)	A non-opioid NMDA receptor antagonist	MDD (TRD)	NCT03051256 (phase 2)
	EVT-101	An NMDA GluN2B antagonist	MDD (TRD)	NCT01128452 (phase 2) (T)
iGluR-AMPA	ORG 24448	an AMPAkine as AMPA receptor potentiators	MDD	NCT00262665 (withdrawn)
			Schizophrenia	NCT00425815 (withdrawn)
	CX516	An AMPA receptor positive modulator	Schizophrenia	NCT00235352 (phase 3) (C)
mGluR or other pathways	N-Acetyl-Cysteine (NAC)	May restore glutamate to its correct levels in the brain	Schizophrenia	NCT02505477 (phase 4)
			MDD (TRD)	NCT02972398
	Pomaglumetad methionil (LY2140023)	Metabotropic glutamate 2/3 receptor (mGluR2/3R) agonist	Schizophrenia	NCT00149292 (phase 2) (C)
			Schizophrenia	NCT01307800 (phase 3) (T)
	JNJ-40411813 (ADX-71149)	mGluR2 positive allosteric modulator	Schizophrenia	NCT01323205 (phase 2) (C)
	AZD-8529	mGluR2 positive allosteric modulator	Schizophrenia	NCT00921804 (phase 2) (C)
	RO4995819 (Decoglurant)	GluR2/3 negative allosteric modulator	MDD	NCT01733654 (withdrawn)
	Basimglurant	mGluR5 negative allosteric modulator	MDD	NCT01437657 (phase 2) (C)
	Diazoxide	Increases glutamate uptake from the synaptic cleft	MDD	NCT02049385 (phase 1) (T)
	Ceftriaxone	Decreasing the amount of extracellular glutamate in brain	Schizophrenia	NCT00591318 (phase 1) (T)

**Figure 2:**
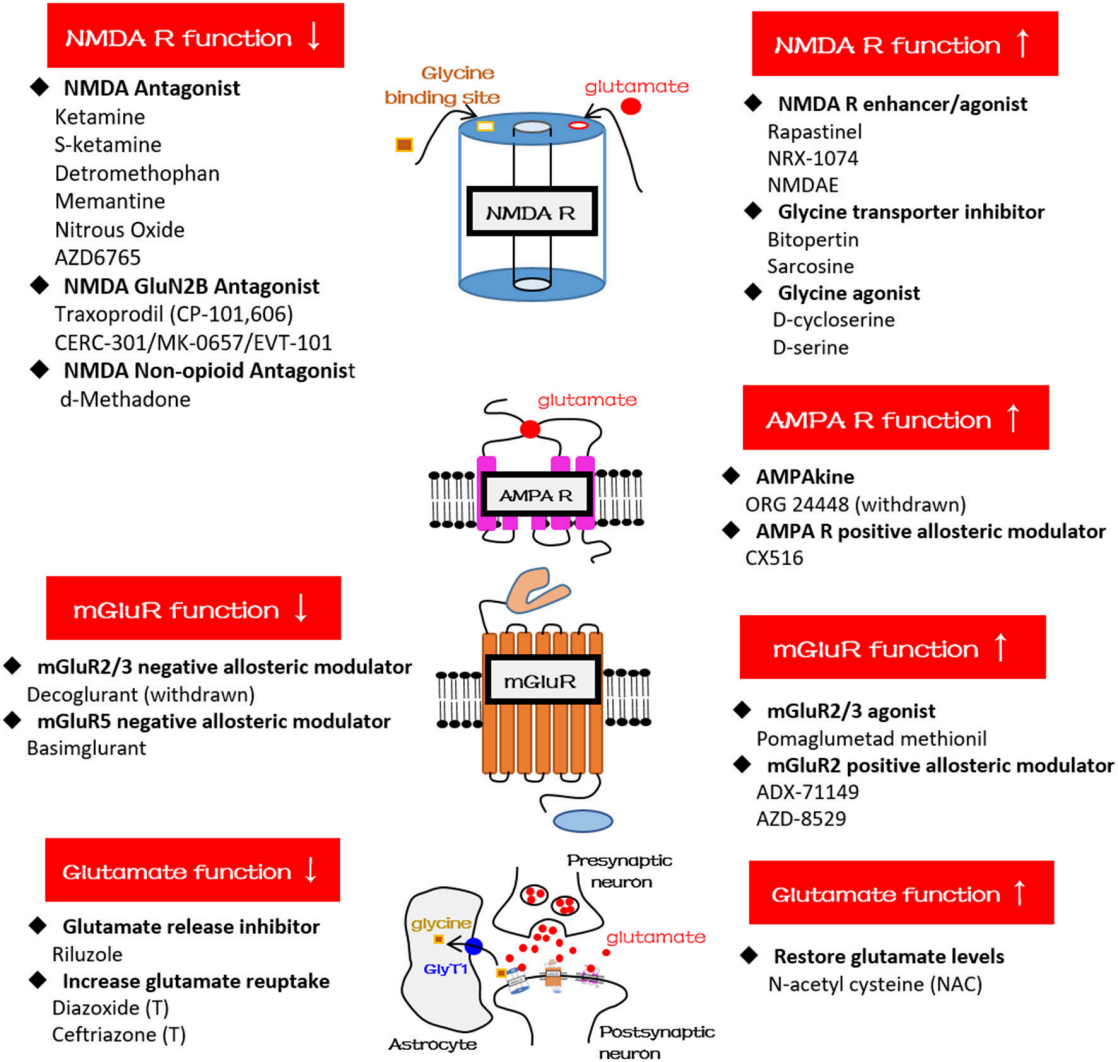
Schematic diagrams and glutamatergic compounds for NMDA receptor, AMPA receptor, mGluR, and glutamatergic neurotransmission, respectively. GlyT1, glycine transporter type-1.

The most attractive compounds are NMDA antagonists because a growing body of evidence has pinpointed glutamatergic dysfunction in the pathophysiology of TRD and demonstrated that the glutamatergic synapses present multiple targets for development of novel antidepressants. For example, ketamine and its S-enantiomer (esketamine) are NMDA antagonists and, when used in a low-dose range, have exhibited rapid antidepressant properties for TRD (70). In a low-dose range, tolerability seems to be acceptable with transient elevation of blood pressure and mild and self-limited psychotomimetic effects (70). Several other compounds, such as dextromethorphan, memantine, traxoprodil, AZD6765, and riluzole, among others (see Table 2 and Figure 2), have similar pharmacological properties and have great potential in treating MDD and BD depression. Using a small sample size (*n* = 14 completed the study), researchers in an open-label study found that dextromethorphan/quinidine (Nuedexta; Avanir Pharmaceuticals, Inc., Aliso Viejo, CA, USA) could decrease depression scores with acceptable tolerability in patients with TRD (71). Traxoprodil, an NR2B subunit-selective NMDAR antagonist, in combination with paroxetine was found to decrease depression scores for patients with TRD (*n* = 30) (72). A single intravenous dose of AZD6765 (a low-trapping NMDA channel blocker) was also found to have rapid but short-lived antidepressant effects in a small trial (*n* = 22) (73). However, longer-duration and larger studies are required to prove clinical efficacy because not all NMDA antagonists possess rapid antidepressant efficacy. For example, an NMDA antagonist, memantine (74), had been shown to lack rapid antidepressant effects. Likewise, riluzole was found to have limited effects for ketamine nonresponders (75). Notably, sarcosine, an NMDA enhancer, had been found to improve depression-like behavior in rodent models and depression in humans (76). Some iGluR NMDA-related compounds are also used for treating schizophrenia (Table 2). For example, d-serine, a naturally occurring NMDAR glycine site agonist, was found to have significant effects on auditory mismatch negativity that correlated significantly with change in symptoms of schizophrenia in a small double-blind crossover trial (*n* = 16) (77). Researchers in a randomized, multicenter, double-blind, placebo-controlled study investigated adjunctive RO4917838 (bitopertin), a selective GlyT1-mediated glycine reuptake inhibitor, in patients with schizophrenia with suboptimally controlled symptoms (*n* = 1,772) and found that the antipsychotic effects were small (mean difference vs. placebo in score, −1.37) and were demonstrated in only one of six active treatment arms (78). Some trials have been withdrawn or terminated (Table 2), including a trial using EVT-101 for MDD, trials using Org 24448 for MDD and schizophrenia, a trial using diazoxide for MDD, and a trial using ceftriaxone for schizophrenia. The reasons may include prominent side effects or a lack of clinical efficacy, so the final outcomes of these clinical trials must be awaited.

Glutamatergic compounds are also used for treating BD depression. For example, researchers in a randomized, double-blind, placebo-controlled study investigating a single intravenous infusion of ketamine (0.5 mg/kg) for BD depression found that depressive symptoms and suicidal ideation significantly improved within 40 min (79). In addition, a small proof-of-concept study investigated the effects of ketamine and d-cycloserine in patients with BD, in which subjects received open-label ketamine hydrochloride (0.5 mg/kg intravenously over 60 min) followed by 8 weeks of adjunctive d-cycloserine (titrated up to 1000 mg/d from a starting dose of 250 mg over 3 weeks) (80). The investigators found that four of seven subjects met remission criteria at 8 weeks. However, a randomized controlled study of riluzole monotherapy (50–200 mg/d) for BD depression was terminated early (Table 2) because of a high number of subject withdrawals and no significant antidepressant effects of riluzole (81).

Although most iGluR NMDA compounds seem to be used for treating MDD (Table 2), iGluR-AMPA and mGluR compounds are generally used for treating schizophrenia (Table 2). For example, CX516 (an AMPA receptor-positive modulator and also the first ampakine) was used for cognitive enhancement in schizophrenia; however, the results appear to be disappointing because CX516 was not effective for cognition or for symptoms of schizophrenia when added to clozapine, olanzapine, or risperidone (82). *N*-acetylcysteine is a widely available dietary supplement that may restore glutamate to its correct levels in the brain and is used for treating cognitive deficits in schizophrenia (Table 2). LY2140023 as an mGluR2/3 agonist was originally found to have significant antipsychotic effects in patients with schizophrenia early in disease or in those previously treated with D2 drugs (83), but investigators terminated another study because LY2140023 failed to achieve significant effects on the overall symptoms of schizophrenia (Table 2).

Hypofunction of NMDARs has been suggested to play an important role in the pathophysiology of schizophrenia, and glutamate PAMs may be effective for treating schizophrenia and related cognitive deficits. However, to date, the results have been inconsistent. For example, authors of a recent meta-analysis that included 17 randomized, placebo-controlled studies (*n* = 1,391) found that glutamate PAMs were not superior to placebo in improving cognitive function in schizophrenia (84). In addition, results of mGluR2 activators for schizophrenia seem to be inconsistent. For example, Eli Lilly's mGluR2/3 agonist (LY2140023) failed to meet the primary endpoints in a phase II trial (85), and another phase III trial was stopped mainly owing to the lack of efficacy; however, JNJ-40411813 (a PAM being developed by Janssen Pharmaceutica NV Beerse, Belgium, and Addex Therapeutics, Geneva, Switzerland) has been shown to have effects on negative symptoms in patients with schizophrenia in a phase II trial (85). Another mGluR2 receptor PAM (ADZ8529; AstraZeneca, Cambridge, UK) failed to separate from placebo in total, negative, and positive symptoms of schizophrenia in a phase II trial (86).

A recent paper published in *JAMA Psychiatry* in which researchers evaluated basimglurant (mGluR5 NAM) for patients with MDD with inadequate antidepressant responses in the current episode found that the primary endpoint (mean change in clinician-rated depression score from baseline to endpoint) was not met, but an antidepressant effect on patient-rated measures was found across secondary endpoints (87). However, an NAM targeting mGluR2/3 (decoglurant) was withdrawn, mainly owing to disappointing antidepressant efficacy (Table 2).

## Conclusion

Although most techniques today indirectly measure glutamatergic neurotransmission *in vivo*, accumulating evidence has revealed that glutamatergic dysfunction plays a crucial role in major psychiatric disorders, such as schizophrenia, BD, and MDD (including TRD). Ketamine, esketamine, and many other pharmacological compounds targeting the glutamate system are available for human trials of major psychiatric disorders. However, longer-duration and larger studies are required to prove their clinical efficacy in different psychiatric diseases.

## Author Contributions

C-TL: conceived and designed the study; C-TL, K-CY, and W-CL: performed the analysis and review; C-TL, K-CY, and W-CL: wrote the paper; C-TL, K-CY, and W-CL: approved the paper.

### Conflict of Interest Statement

The authors declare that the research was conducted in the absence of any commercial or financial relationships that could be construed as a potential conflict of interest.
